# Specialist Financial Counseling for Women Affected by Domestic and Family Violence: Staff and Client Perspectives on an Australian Initiative

**DOI:** 10.1177/10778012241263103

**Published:** 2024-07-25

**Authors:** Natasha Cortis, Ciara Smyth

**Affiliations:** 1 Social Policy Research Centre, 7800 University of New South Wales, Sydney, NSW, Australia

**Keywords:** economic abuse, financial abuse, domestic violence, financial counselor, co-location

## Abstract

This article examines staff and client perspectives on an initiative providing co-located specialist Domestic and Family Violence (DFV) financial counseling in women's legal services. An exploratory mixed-method study in five service locations captured perspectives via a client survey (*n* = 42), online interviews with staff (*n* = 15), and a review of services’ progress reports. For staff and clients, integrating financial counseling into women's legal services contributed to a more comprehensive model of support which helped address the economic harms associated with violence. Findings highlight the benefits of service integration and co-location, which enabled staff to share knowledge and build capacity, and helped improve outcomes for women following violence.

## Introduction

Control over women's finances and other threats to their economic security and self-sufficiency are aspects of domestic and family violence (DFV) that affect women globally. Research has detailed how economic abuse, which incorporates, but is not synonymous with financial abuse, involves a wide range of tactics of economic restriction, exploitation, and sabotage. When employed within heterosexual couple relationships, these tactics are usually levied by men against women, and make it difficult for women to leave (Cortis & Bullen, 2016; Macdonald, 2012). Tactics include withholding or controlling income to increase women's dependence on men; not contributing to household expenses; shifting debt liability to women; denying access to income and assets; and preventing or interfering with women's employment, financial decision-making, and autonomy (Adams et al., 2020; Cortis & Bullen, 2015; Johnson, 2021; Macdonald, 2012; Schrag et al., 2019; Sharp-Jeffs, 2021). Sometimes, economic abuse continues after relationships have ended, as abusers continue trying to control or punish ex-partners, including through non-compliance with debt servicing or child support arrangements (Branigan, 2004; Cook et al., 2023; Kaittila et al., 2024; Krigel & Benjamin, 2021). Further, economic abuse often co-occurs with physical, emotional, and sexual violence, all of which generate significant financial and psychological costs and harms (Adams et al., 2008; Eriksson & Ulmestig, 2021).

Although both men and women may perpetrate economic and other forms of violence, and domestic and family violence may occur within gay, lesbian and bisexual domestic relationships as well as between heterosexual partners, most violence is perpetrated by men, and overwhelmingly, harms are borne by women (Flood et al., 2022, p. 14). In Australia, males are responsible for almost 9 out of 10 homicides; three-quarters of DV perpetrators are male, and males are six times more likely to perpetrate sexual violence and sexual harassment than females (Flood et al., 2022). Reflecting the gendered nature of violence and the consequent focus of victim support services on women, our focus here is on women. Economic abuse, and the economic harms of physical and psychological abuse, reverberate through women's lives for years. Women who have been subject to partner violence are over-represented among government income support recipients (Summers, 2022) and among those accessing ‘Buy Now Pay Later’ or other costly, under-regulated credit options (King, 2023). Women with repeated or prolonged exposure to violence have most ongoing difficulties paying bills and affording food, and consequently, have a greater need for assistance from welfare agencies than others, even long after the violent relationship has ended (Cortis & Bullen, 2015, 2016).

While economic abuse is increasingly recognized as a feature of violent relationships, it remains difficult to identify, measure, and address. Measurement is challenging because economic abuse can involve a wide range of behaviors, it lacks a standard definition, and it may be obscured by other forms of violence (Bullen et al., 2016; Postmus et al., 2020). Furthermore, the normalization of men's economic power, and persistent cultural taboos around discussing household finances, make it difficult for women to recognize that control over money and other resources constitutes abuse. Even when economic abuse is identified, women may not know where to seek assistance, or may choose not to do so (Glenn & Kutin, 2022; Sharp-Jeffs, 2022).

Globally, economic abuse as a form of intimate partner violence has slowly gained visibility and recognition. A multicountry review identified 46 studies in 19 high, middle and low-income countries across six continents that examined economic abuse as a form of intimate partner violence (Postmus et al., 2020). In some countries, governments are trying to monitor it, as a basis for action. The Australian Bureau of Statistics’ Personal Safety Survey, for example, indicates that around 16% of women experience economic abuse at some point throughout their lives (Kutin et al., 2017). Earlier Australian studies similarly found that economic abuse affected between 80 and 90% of women seeking domestic violence crisis services and support (Evans, 2007; Macdonald, 2012), and Glenn and Kutin (2022) found that it was a factor in at least 70% of violent relationships. In the United States, more than three-quarters of survivors seeking support for intimate partner violence have been found to have experienced economic abuse (Johnson et al., 2022). Yet, despite its increasing visibility, this form of abuse is often assumed to be less harmful than physical or sexual violence (Postmus et al., 2020), and policy and practice initiatives to prevent and address it have been slow to develop.

Ideally, comprehensive, population-wide support would work to prevent men from using violent, controlling tactics, and would ensure that women had practical, affordable alternatives to living with violence and did not need to be economically dependent on men (Corrie & McGuire, 2013; Braaf & Barrett Meyering, 2011). To achieve this vision of ending economic abuse, research has underlined the need for large-scale, coordinated change across multiple systems and within the crisis sector (Braaf & Barrett Meyering, 2011; Cortis & Bullen, 2015; Tonkin, 2018). Some strategies to tackle economic abuse have been recommended and reported in the literature (Bullen et al., 2016; Cortis & Bullen, 2016; Hahn & Postmus, 2014; Johnson, 2021; Kintominas, 2019; Kutin, 2019; Warren et al., 2019) Common features include:

- promoting the awareness of the tactics involved in economic abuse;
- building women's financial literacy and financial management skills;
- helping women in violent relationships to build and secure their assets;
- adapting policies of banks and utilities (water, electric, and gas) to address economic abuse, and
- training domestic violence practitioners to work more effectively with survivors around financial issues, including by ensuring safety plans recognize economic needs.

In general, research notes the need to develop and expand these types of initiatives. Indeed, initiatives to address economic abuse remain small and ad-hoc. They are usually delivered as local, individualized, short-term, and pilot interventions, which are not routinely evaluated to determine effectiveness, or are evaluated but with small samples or other limitations (e.g., Warren et al., 2019). To inform more effective responses, more evidence is needed about which initiatives are effective for addressing the economic aspects of gender-based violence, how to take effective initiatives to scale, and ultimately, how to achieve the cultural change necessary to dismantle gendered economic inequalities and harms (Rose et al., 2023).

To this end, Australia has a high-level, government-led framework that sets a long-term vision and plan to address gender-based violence: the *National Plan to End Violence against Women and Children 2022–2032.* It emphasizes the need to build specific capacity to recognize and prevent financial abuse and to address the heightened risks of poverty for victim-survivors. The National Plan expresses commitment to holistic strategies, including working with banks, credit, utility companies, and other services to address debt resulting from coercion, and building capacity by training more financial specialists about the financial tactics of DFV (Department of Social Services, 2022). In practice, however, enacting these commitments is limited by the weak evidence base on effective initiatives to address economic abuse for the diverse range of people it affects. The National Plan notes the need for more evidence about the financial aspects of DFV, and effective ways to respond.

This article contributes by presenting the findings of a mixed-method study of an Australian initiative to address economic abuse. Specifically, it employed specialist DFV financial counselors who were strategically co-located in women's legal services, to provide holistic support to promote the economic capacity and status of women affected by DFV. The initiative was developed by a philanthropic foundation that provided grants to ten Australian women's legal services, enabling them to integrate financial counseling into their existing service mix. Although financial counseling cannot resolve the structural determinants of poverty, inequality, violence, and injustice, it holds promise as a service to help people understand and assert control over their financial situation, and hence, can help support individual women affected by violence and gradually shift gendered patterns of economic disadvantage. Financial counseling is a generalist service primarily set up to help people set budget and financial goals and negotiate bills, debts, and fines, but specialist models of financial counseling have been developed to assist women affected by violence, by providing essential information, support, and advocacy; helping women to understand their rights and navigate consumer and debt issues; and helping women alleviate financial difficulties and build financial capacity following violence.

While financial counseling may look different in different national and local contexts, in Australia, it usually involves assessment, advice, advocacy, education, and psychosocial support. Models of specialist financial counseling grew out of innovative credit counseling programs in the late 1960s and were expanded in Australia in the 1970s when Commonwealth and State and Territory governments allocated short-term funding to services operated by non-government organizations (Jones, 1982). Specialized approaches grew from generic financial counseling models to support cohorts vulnerable to extreme hardship and poverty, including older people, people in rural communities, and women who have experienced DFV (see, for example, West & Ramcharan, 2019). However, inadequate funding support for service delivery continues to impede the growth and maturity of the financial counseling sector and the expansion of specialized approaches (Sylvan, 2019). Many promising initiatives have not been sustained or have been unable to attract the funding needed to operate at scale to meet needs. While the National Plan is geared toward generating the coordinated national action needed to foster longer-term change, philanthropy can play a role by establishing, trialing, and evaluating innovative models. Here, we examine one philanthropic initiative that involved employing and co-locating specialist financial counselors in women's legal services. We first describe the model, then outline the study methodology, and present findings about how the model operated and its impacts on clients and staff. We conclude by discussing the implications of the findings including the challenges of evaluating the initiative and taking successful interventions to scale.

## Study Context

Data were obtained from an exploratory study of five of the 10 services funded by a philanthropic foundation to employ and co-locate specialist DFV financial counselors within women's legal services to support women affected by DFV. Funding documentation outlines the objectives of the initiative in contributing to the safety and well-being of women who have experienced or are experiencing DFV; helping them to develop knowledge about the nature of economic abuse and their rights, responsibilities, and options; developing capability and confidence to move toward financial independence and stability; and promoting economic justice through advocacy, including by removing unfair debts.

The initiative was implemented in mid-2020, during the first year of the COVID-19 pandemic, although it was not specifically designed as a pandemic response. At the time, advocates in the DFV sector recognized that stay-at-home orders were leading to increased violence against women, services were finding new ways to respond, and pandemic-related increases in financial stress underlined the acute need for financial support for women (Cortis et al., 2021; Morgan & Boxall, 2022; Smyth et al., 2021). The initiative was conceived to firstly improve outcomes for women dealing with the consequences of economic abuse and secondly, to build capacity among co-located staff through the integration and co-location of services.

Each of the five services operated in different Australian jurisdictions. The services had different structures and histories but all established foundations in place to support women with the legal needs associated with violence (e.g., family violence restraining orders, family law matters, and criminal injuries compensation claims relating to family or sexual violence) along with other services (e.g., advocacy for law and policy reform, community loans, and community education). Each service received funding to employ a financial counselor, who was to work in an integrated way with legal services to meet women's needs more comprehensively and build service capacity to address economic abuse. While accreditation of financial counselors in Australia ensures quality standards, a uniform program model was not used across the sites. Rather, the funding allowed for adaptation to meet local needs. Prior to receiving the grant, four of the five services had not delivered financial counseling (and had referred out), while one had existing financial counseling capacity and used the funds to develop specialization relating to economic abuse.

Across the services, financial counseling was provided to women via drop-in services, outreach, and internal and external referrals. Internal referrals were from legal practitioners assisting women with legal matters and external referrals were from local service networks such as housing and tenancy organizations, Aboriginal organizations, alcohol and other drug services, and via utility companies and the National Debt Helpline. Financial counseling was provided in person and over the phone. Depending on service demand, financial counselors would try to contact clients as soon as possible and schedule appointments within 3–4 days. There was no limit to the number of times a client could contact a financial counselor and the total average duration of client engagement varied considerably depending on the complexity of their circumstances, ranging from 1–2 hours to 20 hours.

Financial counselors were nationally accredited and had a specialized understanding of DFV. They helped women by assessing their financial situation, assisting with budgeting and goal setting, and managing debt, bills, fines, and scams. They also helped women develop knowledge about their financial rights and financial abuse to build the capability to move toward financial independence and stability. The co-location of financial and legal support was intended to improve service systems and accessibility while promoting professional collaboration. The co-location of specialist DFV financial counseling within women's legal services acknowledges the multifaceted nature of violence against women and highlights a form of abuse that is often less visible.

## Methods

The study focused on five of the ten funded services. Sites were selected by the funder prior to engaging the university research team, not randomly. The five selected sites had been able to recruit financial counselors and commence service delivery sooner than other sites and the funder considered them to have the capacity to participate in the study. As such, some positive selection bias was likely.

Key research aims were to establish whether and how having a specialist DFV financial counselor located in women's legal services resulted in improved outcomes for women and improved service responses. The study sought client and staff perspectives on how the specialist financial counseling component was working within the broader women's legal service. The mixed-method design consisted of interviews with financial counselors, managers, and legal practitioners (*n* = 15); an online client survey (*n* = 42); and a review of services’ biannual progress submitted to the funder. The approval to conduct the research was granted by the University of New South Wales Human Research Ethics Committee (approval number HC220439).

*Staff interviews* aimed to capture perspectives on having a specialist financial counselor in the legal centers’ staff mix and whether it contributed to improved client outcomes. Staff were invited to participate via an email invitation from the research team sent by the service manager to the staff. Three staff per service were invited to participate in an online interview and fifteen staff were interviewed in August and September 2022. The staff interviewed included financial counselors in each site, along with business managers, legal staff, and administrative staff, the mix of which differed by site. Interviews were recorded and transcribed in full, or detailed notes were taken, before content was analyzed thematically using iterative categorization to code and summarize (Neale, 2016). The codes captured service operations and observed client outcomes and benefits and challenges to integrating financial counseling within their service.

*An online client survey* was developed to capture clients’ perspectives on receiving specialist DFV counseling. Engaging clients affected by DFV in research is challenging due to the experiences of poverty, financial and housing instability, and safety risks associated with DFV. Therefore, the research design prioritized client safety. An ethical survey protocol was designed which involved service staff informing clients about the survey, which was live in November 2022. Where considered safe to do so, staff in the five services sent survey information and a link to women who had used the specialist financial counseling service in the last 12 months. Contact with the women was via email, phone, or SMS. Although services forwarded the survey information to clients, all survey completions went through to the research team, not the service, to ensure confidentiality. Clients who completed the survey could enter a prize draw for one of the five $100 shopping vouchers, nevertheless, it remained difficult to obtain high completion numbers.

In total, 42 surveys were completed across the five services, although numbers ranged from 2 (Service 1) to 14 (Service 4). The research team relied on the services to distribute the survey so that clients received it from a trusted source in accordance with their communication preferences and safety needs, and to ensure women's contact details were not shared with the researchers. Recognizing that services had limited administrative resources, they were not asked to report on the number of clients invited to complete the survey, and as a result, a meaningful response rate could not be determined. The sample is likely skewed to clients who were easiest to reach, had maintained contact with the service, and were not currently experiencing crisis.

Most participants were aged in their 30s (17 or 41%) or 40s (14, or 33%). One in three (14 or 33%) reported having caring responsibilities, and one in seven (6 participants or 14%) identified as a person with a disability. Seven participants (17%) identified as a person from a culturally and linguistically diverse background and five people (12%) identified as Aboriginal and/or Torres Strait Islander. Based on administrative reports on client characteristics from services, the proportion who completed the survey from these groups was lower than their makeup among those who received services. Study participants are also likely to be among clients who are better connected to services. Indeed, many had high levels of engagement, with half of the survey participants (21 or 50%) having met or spoken with a financial counselor more than five times. Survey analysis involved descriptive statistics for closed survey questions. Open-ended questions were anonymized and analyzed thematically.

*Progress reports* were provided by services to the funder every six months and reports for the five study sites were reviewed. They provided information on grant expenditure, and reported on operations, including the number of clients seen by the specialist financial counselor, client characteristics, any pressures on the services, and reasons for serving fewer clients than anticipated, if this occurred in the period. The progress reports also captured counts of client demographics, types of financial difficulties recorded, casework activities completed, and allowed services to provide examples of ‘case studies’ of women they had assisted. Some reports included other information, such as the number of appointments held and total client savings achieved by the program. While these reports do not provide a comprehensive indication of the nature or extent of the outcomes resulting from the initiative, the contextual information they provided was helpful for understanding how services were implemented and integrated specialist financial counseling and the pressures facing clients and communities.

## Findings

First, we present client and service characteristics, setting the scene for exploring the ways the financial counseling component worked within the broader women's legal service, and its effectiveness, based on staff and clients’ views.

### Financial Counseling and Client Demographics

The five services provided financial counseling to over 2500 women between mid-2020 and the end of 2022. Women accessing financial counseling had highly complex financial needs associated with experiencing DFV indicating appropriate targeting of the financial counseling service. Although data were incomplete, most clients had experienced economic abuse or were described as ‘at risk’ of economic abuse. 60% of clients were sole parents with children and 76% had weekly incomes under $800 (Australian dollars). Around a quarter spoke a language other than English at home (26%) and 21% were Aboriginal and/or Torres Strait Islanders. Many services noted the increased complexity of women's needs, due to rising cost-of-living pressures affecting Australian communities. During 2022, women had been affected by inflation and rising food and household costs, as well as housing stress due to interest rate rises and a lack of affordable rental options, which were perceived by services to be making it more difficult to leave abusive relationships.

The specialist financial counselors provided a range of support related to economic abuse. Most commonly, they supported clients whose financial difficulties related to rent or mortgage arrears; household, credit card, and utility debts; Centrelink (social security) debts; and unpaid fines. Women had become more reliant on credit cards and for many, debt was spiraling from the increased use of *Buy Now, Pay Later* credit options, including for food and household bills. Most commonly, specialist financial counselors’ casework activity involved providing clients with assistance and referrals, liaising with companies to remove and settle debt, gaining access to debtor's hardship programs, supporting women to self-advocate and access entitlements, obtaining loans and support packages, and developing achievable payment plans.

Each service began with different levels of capacity to deliver specialist financial counseling and faced different pressures. One operated in line with planned client numbers and two served fewer clients than anticipated due to difficulties recruiting a financial counselor. Reasons for serving fewer clients than anticipated included that referral pathways into the service were underdeveloped, because clients had highly complex circumstances, and because of client non-attendance and engagement difficulties. Two services faced significant demand pressure and served more clients than planned, and one of these described a long waiting list and having to pause referrals when appointment times were all booked, with staff able to prioritize only the most urgent cases.

### Promoting Women's Financial Empowerment and Safety: Staff Perspectives

At a high level, the outcomes that clients were seeking, and that staff supported them to achieve, related to financial empowerment and safety. Specialist financial counselors described how their work involved working closely to engage vulnerable clients, help break harmful cycles, and achieve the security and confidence needed to live independently and remain safe, without the need to return to a perpetrator or enter unsafe relationships in the future. Counselors emphasized the way the initial phase of services involved building safe, trusting communication, working on immediate needs (such as applying for emergency government payments or food vouchers), compiling information (such as paperwork relating to debts or assets), and supporting women to set practical goals. Usually, women's goals included: securing accommodation so they could establish their own households; being in a position to apply for and undertake paid work, and removing and reducing unfair debts resulting from violence, to get a ‘clean start’ financially. To achieve these goals, financial counselors negotiated with banks, utility companies, and other services and sought to relieve debts resulting from financial abuse. They helped women build financial capability for the long term, including helping them to establish budgeting strategies to achieve their financial goals. Counselors understood the high costs women incurred because of domestic violence, including the need to relocate with children to new communities away from perpetrators.

Importantly, while the specialist financial counseling initiative sought to empower clients financially and promote women's safety, the staff interviewed recognized that it could not address all risks and meet all needs. They considered specialist financial counseling to be critical, but more comprehensive interventions to prevent and address the financial impacts of violence are needed. These include a strengthened social security safety net with women's needs at the center and improved consumer credit regulation.

### Integrating Financial Counseling with Women's Legal Services

Integrating financial counseling within the existing women's legal services built capacity among legal practitioners, managers, and other colleagues to understand the gender-based financial impacts of domestic violence in all five sites. Financial counselors helped their colleagues to understand how the financial issues of clients were intertwined with legal, health, housing, employment, and other issues they faced following the violence. They helped colleagues to understand why women affected by violence may not answer the phone or provide documents on time. They improved legal practitioners’ understanding of clients’ communication preferences, so that they could tailor their engagement accordingly, to ensure accessibility and safety. Staff recognized that financial counselors had expertise that the service could not otherwise provide and that the initiative provided the capacity to prioritize financial issues. One stated, for example, that “We’re funded to be a short, sharp intervention and we wouldn’t have time to do budgeting or any of those things with clients.” (Domestic violence worker, service 3)

Legal practitioners specifically valued specialized financial counseling to enhance their capacity to work effectively with women affected by violence and to provide alternative avenues to achieve justice. Lawyers appreciated how financial counselors could provide clients with financial strategies, while they awaited court outcomes. One felt she was “a much better lawyer” after learning how financial counselors support clients, and shared reflections on the way the collaboration changed how she thought about achieving outcomes for clients:

Sometimes lawyers, for example, just want to fix the issue. They just want the court matter to finish, and they'll tell a client to agree to particular things because it's in their best interests. When I work with financial counselors, I realize [I need] to look at the situation from their side and say well [the strategy] is not in the client's interest, because they’ll agree to an outcome that is not sustainable. (Lawyer, Service 1)

All lawyers recognized financial counselors’ skill in addressing clients’ urgent financial issues to prevent the escalation of debt while awaiting legal outcomes, and all financial counselors valued being able to collaborate with lawyers and give and get advice to address client issues, noting that this collaboration was supported by protocols to ensure confidentiality and maintain professional boundaries.

Lawyers in the service will give me a quick call … I’m able to answer that question … so they’re not wasting their time having to source that information. It's an easy process for me because I can go directly to the lawyer and ask, “Where are we at with this?” … Just that free flow of communication … If you’re all working … toward the same goal, and you’ve got a multi-pronged approach to it, it's more beneficial [ for the client]. (Financial counselor, Service 2)

Staff valued how the co-location of the specialist financial counselor within the legal service enabled them to draw on a multidisciplinary team to deliver more comprehensive support which improved client engagement, expanded the capacity to address a wider range of client needs, and reduced the need to disrupt support by referring externally:

Having a financial counselor come on board, it's actually been quite extraordinary because debt issues, loan issues, just anything really, fines, Centrelink, all these card debts, credit card debts, it's really something that we, due to capacity issues, couldn’t have really sat down and done a lot. We may have taken one or two on, but we would’ve probably seen where we could refer them before we had a financial counselor. (Manager, Service 2)

The co-located model was also valued as a better way to deliver support, which provided efficiencies for clients, who could access multiple types of support in one place. As Carrie, a financial counselor explained: “We have a lot of things under one roof, and this is very beneficial, especially when clients are very traumatized. They have, in our office, possibly four people to talk to. Imagine if they had to run to four offices?” (Service 4)

### Sustainability Challenges

While welcoming the initiative, staff expressed concern about its sustainability. Managers described how specialist financial counselors were particularly difficult to recruit, and underlined the need for longer employment contracts and sustained funding. Adequate support for financial counselors was a critical ingredient of success, and staff recognized the importance of supervision, debriefing, professional connection, and knowledge sharing to enhance effectiveness.

We’re lucky that we can talk to each other. We have a debriefing. We have supervision. I check in with people every week or every fortnight just to see how they’re doing. People come to me if they’re really struggling or finding the stories quite difficult. (Manager, Service 1)

Staff were concerned about the uncertainty around ongoing funding beyond the grant period. They spoke about the growing demand for financial counseling, the high levels of need in the community, and long waiting lists. Many staff were worried about the services’ ability to retain specialist staff and continue offering financial counseling beyond the grant period. Many saw a need for larger financial counseling teams to meet the demands and prevent professional isolation. Suggestions included: expanding financial counseling teams; employing financial capability workers to work alongside financial counselors; and collaborating with external services (e.g., hosting government social security officers on-site) to expand reach and enhance team capacity and knowledge sharing. Staff recognized that the long-term success of the initiative was dependent on having a sustainable funding model and that extending grants or attracting government funding were potential solutions. However, the lack of an in-built mechanism for transitioning to an alternate funding model posed challenges for managers in making decisions about the future sustainability of the model. Additionally, financial counselors expressed uncertainty about their future and whether they should seek new positions.

### Perceptions of Quality and Outcomes

Clients provided positive feedback about specialist financial counseling. In addition to receiving help relating to DFV directly, surveyed clients most commonly reported receiving help relating to banks, such as credit card debt or loans (45%); debt collectors (45%); utility companies, including energy, phone, and internet providers (40%); Centrelink (31%); and accessing crisis payments or emergency food relief (26%). Many also received help with housing issues, i.e., assistance relating to a landlord, housing authority, or crisis accommodation (19%) or help to set up a new home (12%); help to access and liaise with lawyers (14%); and help relating to child support and accessing health or mental health services (7%).

With the caveat that survey respondents may not represent all clients, participants were overwhelmingly positive about their experiences of engaging with the financial counselor. When given five statements relating to service quality and asked to indicate whether they agreed or disagreed, all participants said they agreed that “The financial counselor understood my family circumstances”, “The financial counselor understood my financial situation and needs”, “My background or identity was respected”, “The options offered were realistic and practical”, and “I would recommend the financial counseling to others”. While this is consistent with the positive experiences of the specialist financial counseling and its ability to meet needs, it also likely reflects participant selection biases. As noted above, these are difficult to avoid in research with DFV survivors recruited via services. While recruitment via services offers a safe and ethical approach, it necessarily prioritizes those with higher service engagement, and so risks excluding those whose needs may have been less effectively met.

When asked if the service helped with various things, ‘a lot’, ‘a little’ or ‘not at all’ (Table 1), responses clustered in the positive response categories. Almost all said the specialist financial counseling had helped ‘a lot’ with accessing information and services (98%), with little variation across sites or groups of women. Almost as many (93%) said it had helped ‘a lot’ in making them feel capable and confident. Most (85%) said that the service helped ‘a lot’ with prioritizing safety, with a further 12% saying it helped ‘a little’. Large proportions (83%) said it helped ‘a lot’ to minimize future hardship and to manage bills, debts, or fines (also 83%). Slightly fewer said it helped with paperwork ‘a lot’ although many others (17%) said it helped with this a little. Although addressing financial abuse was a core aim of the program, not all felt it was fully achieved: around three quarters (76%) said it helped with this ‘a lot’ while a further 22% said it helped with this ‘a little’.

**Table 1.** Client Ratings of How Much Services Helped With Various Issues (*n* = 42).

	A lot (%)	A little (%)	Not at all (%)
It helped me access information and services	98	2	0
It helped me to feel capable and confident	93	7	0
It helped me to prioritize my safety	85	12	2
It helped me to minimize future hardship	83	17	
It helped me manage bills, debts, and fines	83	15	2
It helped with paperwork	78	17	5
It helped to address financial abuse	76	22	2

When asked about the support they received, several clients provided brief positive testimonials about the service in general, commenting, for example, “brilliant service & outcome”, “excellent help”, and a “wonderful service. I wish it was more available”. Reflecting the limitations of surveys compared with qualitative methods which provide opportunities for interviewers to probe, clients did not consistently elaborate. However, among the more detailed comments, women usually referred to the quality of the service, underlining the importance of the individual financial counselor they worked with, for example: “My counselor was amazing and did everything to help me”. The skills and professionalism of the financial counseling staff were particularly appreciated.

In terms of outcomes, several comments focused on the impact of the counseling on their financial situation, and mentioned the help they received in relation to debts, Centrelink, and preventing property repossessions and homelessness: “My partner left me in lots of debt and the service was able to ask the creditors to waive the debts. I am very happy with the outcome” (Service 3). Importantly, the service helped to expand women's horizons and suggest practical options: Another client explained, for example: “She gave me ideas I had not thought about. This is a really important service”. (Service 3)

Financial counseling was also described as transformative for women and their families, describing, for example, that it: “Has been life changing helping me to manage debt related to a DV relationship with my ex and allowing me to restart my life for my young children” (Service 5). Another participant explained:

[My worker] has changed my life and the difference honestly to my life and my children's lives has kept us safe and off the streets. Without the help of [this service], I would have been homeless, but worse I may have gone back to my extremely dangerous situation just to keep a roof over our head. (Client, Service 4)

Reflecting on clients’ positive experiences of the service, few offered suggestions about how the specialist financial counseling initiative could be improved. Many simply reiterated their appreciation for the support they received, while others emphasized the importance of keeping the service operating. Being the last question in the survey, lack of suggestions about service improvements may reflect satisfaction and gratefulness for the assistance, but the small number of respondents entering comments may also indicate survey fatigue, although the survey was kept relatively short. It may, however, also reflect the difficulty of engaging harder-to-reach women affected by violence, whose needs may be less well met by service systems, which highlights a general challenge for research and evaluation with women affected by violence.

## Concluding Discussion

Economic abuse may be overshadowed by other, more visible tactics of violence, such as physical abuse, but it is increasingly recognized as a feature of violent relationships that generates multiple harms and continues to affect women post-separation (Bullen & Cortis, 2016; Kaittila et al., 2024; Postmus et al., 2020). This article contributes to growing evidence about effective initiatives to address economic abuse. Such initiatives are urgently needed as the impacts of economic abuse reverberate throughout women's lives and across generations. Specifically, the findings demonstrate how embedding specialized practitioners in existing services can help address economic abuse. It provides a case study of how integrated approaches for supporting women affected by DFV can support better client outcomes, including by building capacity among service providers who benefit from learning from other, co-located professionals (Mundy & Seuffert, 2021; Rizo et al., 2022; Rodgers et al., 2023).

While the initiative was not operating at the scale needed to make a difference at the population level, findings show that the integration of specialist financial counselors in existing women's legal services offers a promising model for addressing economic abuse and the financial harms associated with violence. The model enabled a more comprehensive model of support that was valued by staff and clients. Financial counselors, legal practitioners, and managers felt empowered to address a wider range of client needs; and found themselves better able to promote women's safety, independence, and ability to move forward with their lives following the experiences of violence. Co-locating financial counselors within women's legal services deepened their understanding of the gender-based financial impacts of domestic violence among legal practitioners, managers, and other professionals, building their capacity to identify economic abuse and respond effectively. Correspondingly, financial counselors valued being able to collaborate with lawyers, and share advice and strategies to address client issues. Clients also attested to positive impacts. They valued being able to access a financial counselor and felt their counselor delivered practical support that made a real difference to their circumstances.

Inevitably, however, the study has some limitations. While the evidence collected provided compelling insight into the positive impact of the integrated approach and informed the decision to extend the service, the available evidence was far from ideal. As initiatives to address economic abuse develop, more sophisticated evaluation approaches will be needed, and quality indicators should be selected and embedded from the outset. With this initiative, the research team was engaged after the financial counseling services had been established and there was no opportunity to embed ongoing data collection in service operations. Information from clients was retrospective, with no measures to assess change available from clients prior to receiving the service. Indeed, existing measures of economic abuse tend to be designed for clinical purposes or to help understand the nature of abuse experienced (e.g., Adams et al., 2008) rather than for monitoring program impact. Moreover, it is difficult for practitioners to prioritize the collection of baseline data for evaluations when they need to focus on initial engagement and crisis support.

Staff explained that comprehensive feedback was not always available to them about the impact of their work, especially where client engagement was intermittent or disrupted. Sometimes staff only became aware of the impact of the service on clients by chance, by running into a former client months later for example. Debt alleviation data offers potential evidence of impact, and services attempted to record and monitor the total value of debt successfully removed from clients; however, this information proved patchy and was highly contested. Staff and managers tended to consider it a poor indicator of service effectiveness and they noted that it was sometimes not apparent until long after the period of service engagement, if at all. It was not seen to reflect the ‘softer’ financial capability skills practitioners tried to build to empower women, which while strengthened through the financial counseling service, may not immediately result in reduced debt. Changes in debt figures could also be small for financially disadvantaged women and could misrepresent significant change in people's lives.

The assessment of the impact of the initiative was further limited due to the types of evidence available from clients. The initiative sought to support women who are particularly difficult to engage due to the experiences of poverty, stress, and financial and housing instability, as well as the safety risks associated with DFV. These clients experience ongoing challenges and may have limited time and energy to participate in research for a range of reasons, which may reduce response rates. Practical challenges include a lack of reliable access to mobile phones or computers to answer calls or complete surveys, or concerns about using devices for the purpose of providing information about their service use and perspectives, due to technological surveillance and safety risks. In fact, before even getting to the evaluation stage, Warren et al. (2019) reported challenges in recruiting women to participate in a financial literacy pilot in women's refuges, with just 11 completing the curriculum rather than the intended 48 participants. For our study, ethical research principles and client safety were paramount. As such, the small sample of survey participants is unlikely to reflect women in the full range of circumstances or experiences of service delivery. Those invited to complete the survey were considered contactable by service staff and were not expected to be experiencing urgent crises, so were likely to be less vulnerable than others. Results are, therefore, likely to have some positive bias as those selected and who opted in to participate were more likely than others to have favorable experiences of the service.

Notwithstanding these limitations, the article provides insight into a specialist DFV financial counseling initiative that was considered effective by those involved. The co-location of specialist DFV financial counseling services in women's legal services was well received by clients and practitioners. It built practitioners’ capacity to address economic abuse and the financial harms experienced by women survivors of DFV, and clients reflected positively on the service.

While financial counseling can help women develop helpful strategies, it cannot address all of the women's financial needs associated with DFV, nor can it address escalating cost-of-living pressures, or poor regulation of *Buy Now Pay Later* credit which can contribute to a spiral of debt. More comprehensive, larger-scale interventions and advocacy are needed, including strengthening the social security safety net and improving credit regulation. Governments also need to monitor innovation and identify opportunities to expand promising financial counseling programs and to ensure they better meet the needs of diverse and historically underserved groups. Ideally, the experiences of diverse groups of clients and the financial counselors who support them should inform program expansion.

More fundamentally, interventions to promote women's financial capacity are not the answer to ending the problem of economic abuse, which often co-occurs with physical, emotional, and sexual violence. Addressing the drivers of gender-based violence remains a paramount global problem demanding significant national and local resources and responses. Achieving change ultimately requires a reframing of the issue “to put perpetrators in the picture and to focus more on preventing and reducing the perpetration of abusive behaviors” (Flood & Dembele, 2021). It requires men to play a role in shifting the harmful beliefs and culture that drive violence against women. Until the social conditions that produce perpetrators change, however, effective system-level responses, including integrated service delivery, are needed to address the multifaceted needs of victim-survivors of male violence.
